# Supplementary material for: Fast nonlinear integration drives accurate encoding of input information in large multiscale systems
Source: Commun Phys. 2025 Nov 18;8(1):437. doi: 10.1038/s42005-025-02339-z (PMC12626883; doi:10.1038/s42005-025-02339-z)
Supplement: Supplementary file 2 — Supplementary Information [file 42005_2025_2339_MOESM2_ESM.pdf]

# Supplementary Information: "Fast nonlinear integration drives accurate encoding of input information in large multiscale systems"

Giorgio Nicoletti[1,2] and Daniel Maria Busiello[3,4]
[1] *Quantitative Life Sciences section, The Abdus Salam International Centre for Theoretical Physics (ICTP), Trieste, Italy*
[2] *ECHO Laboratory, École Polytechnique Fédérale de Lausanne, Lausanne, Switzerland*
[3] *Department of Physics and Astronomy, University of Padova, Italy*
[4] *Max Planck Institute for the Physics of Complex Systems, Dresden, Germany*

## S1. SUPPLEMENTARY NOTE 1: MULTILAYER MODEL WITH NONLINEAR INTERACTIONS BETWEEN UNITS

We consider a stochastic system whose degrees of freedom (dofs) can be partitioned into $N$ components (or units for now on), each containing $M_\mu$ variables that evolve with a shared timescale $\tau_\mu$, for $\mu = 1, \dots, N$. These partitions can be viewed as different units or layers in a multilayer network, so that the $i$-th node in the $\mu$-th layer describes a continuous stochastic variable $x_\mu^i$, with $i = 1, \dots, M_\mu$. Thus, the dimensionality of unit $\mu$ corresponds to the number of nodes $M_\mu$ in the corresponding layer, with nodes representing its interacting internal degrees of freedom. We will often refer to $x_\mu^i$ as the activity of the node, in reminiscence of models for the dynamics of neural networks, even if this framework is amenable to describe a vast variety of biological and artificial systems, as discussed in the main text. The multilayer network is described by an adjacency tensor $A_{\mu\nu}^{ij}$, which measures the strength of the interaction going from the node $x_\nu^j$ to the node $x_\mu^i$. Hence, the matrix $\hat{A}_{\mu\mu}$ describes the interactions between the nodes of the $\mu$-th unit, and $\hat{A}_{\mu\nu}$ describes the interactions between unit $\nu$ and $\mu$. For stability, we take the self-interactions to be $A_{\mu\mu}^{ii} = 1$.

In full generality, we can describe the dynamical evolution of the system by the set of Langevin equations

$$\tau_\mu \dot{x}_\mu^i = -\sum_{j=1}^{M_\mu} A_{\mu\mu}^{ij} f_\mu\left(x_\mu^j\right) + \sum_{\nu\neq\mu} g_{\mu\nu}\phi_{\mu\nu}\left(A_{\mu\nu}^{i,1}, \dots, A_{\mu\nu}^{i,M_\nu}; x_\nu^1, \dots, x_\nu^{M_\nu}\right) + \sqrt{2D_\mu^i \tau_\mu}\xi_\mu^i \tag{S1}$$

where $f_\mu$ is a generic activation function characterizing the intra-unit interactions between nodes, $\phi_{\mu\nu}$ is another activation function dictating the inter-unit interactions from nodes in unit $\nu$ to the node $x_\mu^i$, $g_{\mu\nu}$ is the corresponding interaction strength, $D_\mu^i$ a constant noise strength, and $\xi_\mu^i$ are independent white noises. In the main text, we introduced $\boldsymbol{\phi}_{\mu\nu}$, defined as $\boldsymbol{\phi}_{\mu\nu}(\hat{A}_{\mu\nu}; \mathbf{x}_\nu) \equiv \phi(A_{\mu\nu}^{i,1}, \dots, A_{\mu\nu}^{i,M_\nu}; x_\nu^1, \dots, x_\nu^{M_\nu})$. In other words, the dependence on the node is reflected in the dependence on matrix elements and components of $\mathbf{x}_\nu$, while the functional form of the activation function stays unchanged. In this work, we set $f_\mu(z) = z$, so that, in the absence of inter-unit interactions, the local dynamics of a unit corresponds to an analytically solvable Gaussian process. Notice also that, in this picture, inter-unit interactions can link the dynamics of multiple nodes belonging to different units and, therefore, evolving on different timescales. Unless otherwise specified, we also take $D_\mu^i = 1$ for simplicity.

We focus on two different kinds of activation functions between the units. In the first case, we consider the coupling between the units to be implemented as a nonlinear summation, i.e.,

$$\phi_{\mu\nu}^{\rm ns}\left(A_{\mu\nu}^{i,1}, \dots, A_{\mu\nu}^{i,M_\nu}; x_\nu^1, \dots, x_\nu^{M_\nu}\right) = \frac{1}{M_\nu}\sum_{j=1}^{M_\nu} A_{\mu\nu}^{ij} \tanh x_\nu^j \tag{S2}$$

where tanh plays the role of a nonlinear activation function. In this case, the activities of the nodes of the $\nu$-th unit are first nonlinearly transformed and then linearly averaged to obtain the overall interaction with $x_\mu^i$. In the second case, instead, we consider an activation function implementing a nonlinear integration of the whole unit activity, i.e.,

$$\phi_{\mu\nu}^{\rm int}\left(A_{\mu\nu}^{i,1}, \dots, A_{\mu\nu}^{i,M_\nu}; x_\nu^1, \dots, x_\nu^{M_\nu}\right) = \tanh\left(\frac{1}{M_\nu}\sum_{j=1}^{M_\nu} A_{\mu\nu}^{ij} x_\nu^j\right) . \tag{S3}$$

In Eq. (S3), the overall activity of unit $\nu$, defined as the average activity weighted on the corresponding interactions with $x_\mu^i$, is *nonlinearly integrated*, i.e., it enters in the activation function as a whole. These two choices have been employed in several contexts, particularly in reservoir computing [1] and more in general in random recurrent neural

networks [2–6]. However, they lead to qualitatively different dynamics and deeply affect dependencies between the units.

In general, solving Eq. (S1) amounts to solving the corresponding Fokker-Planck equation

$$\frac{\partial}{\partial t} p_{1,\ldots,N}(\mathbf{x}_1, \ldots, \mathbf{x}_N, t) = \sum_{\mu=1}^{N} \frac{1}{\tau_\mu} \mathcal{L}_\mu p_{1,\ldots,N}(\mathbf{x}_1, \ldots, \mathbf{x}_N, t) \tag{S4}$$

where $\mathbf{x}_\mu = (x_\mu^1, \ldots, x_\mu^{M_\mu})$, $p_{1,\ldots,N}(\mathbf{x}_1, \ldots, \mathbf{x}_N, t)$ is the multilayer joint probability - with each layer representing a specific unit $\mu$ - describing the probability of the activity of all nodes at time $t$, and $\mathcal{L}_\mu$ is the Fokker-Planck operator of unit $\mu$:

$$\mathcal{L}_\mu = \sum_{i=1}^{M_\mu} \frac{\partial}{\partial x_\mu^i} \left[ \sum_{j=1}^{M_\mu} A_{\mu\mu}^{ij} x_\mu^j - \sum_{\nu \neq \mu} g_{\mu\nu} \phi_{\mu\nu} \left( A_{\mu\nu}^{i,1}, \ldots, A_{\mu\nu}^{i,M_\nu}; x_\nu^1, \ldots, x_\nu^{M_\nu} \right) + D_\mu^i \frac{\partial}{\partial x_\mu^i} \right] . \tag{S5}$$

Eq. (S4) is a highly nonlinear equation in $\mathbf{x}_\mu$ and thus solving it exactly is a formidably challenging task. However, we will exploit the fact that interactions within a unit are linear to obtain an analytically tractable factorization of the nonlinear joint probability $p_{1,\ldots,N}$ in a timescale-separation limit.

## S2. SUPPLEMENTARY NOTE 2: INPUT-OUTPUT SYSTEMS AND MUTUAL INFORMATION

A relevant class of systems (see main text for references) is characterized by the presence of an input unit $I$ which evolves independently on the rest of the system - so that $A_{I\nu} = 0$ for all $\nu$ - and an output unit $O$, whose nodes are not a source of any interactions to other units - $A_{\nu O} = 0$ for all $\nu$. These systems exhibit a hierarchical structure allowing for an clear identification of the signal to be read (the input) and the variables that encode it (the output). In particular, we are interested in computing the mutual information between the input and the output, namely

$$\begin{aligned} I_{IO} &= \int d\mathbf{x}_I d\mathbf{x}_O \, p_{IO}(\mathbf{x}_I, \mathbf{x}_O) \log_2 \frac{p_{IO}(\mathbf{x}_I, \mathbf{x}_O)}{p_I(\mathbf{x}_I) p_O(\mathbf{x}_O)} \\ &= H_O - H_{O|I} \end{aligned} \tag{S6}$$

where $H_O$ is the differential entropy of the output and $H_{O|I}$ the conditional entropy,

$$H_O = -\int d\mathbf{x}_O \, p_O(\mathbf{x}_O) \log_2 p_O(\mathbf{x}_O), \qquad H_{O|I} = -\int d\mathbf{x}_I d\mathbf{x}_O \, p_{IO}(\mathbf{x}_I, \mathbf{x}_O) \log_2 p_{O|I}(\mathbf{x}_O|\mathbf{x}_I) .$$

The mutual information quantifies the dependencies between the output and the input units in terms of how much information they share, Specifically, it captures the reduction of the uncertainty in the output, quantified by its entropy, once the input is known. Notice that the expression in Eq. (S6) holds for any system even without a hierarchical structure, however the identification of inputs and outputs might become more complex, eventually leading to ambiguity in the interpretation of the results.

For what follows, it will be useful to rewrite the mutual information in terms of the function

$$h_{O|I}(\mathbf{x}_I) = -\int d\mathbf{x}_O \, p_{O|I}(\mathbf{x}_O|\mathbf{x}_I) \log_2 p_{O|I}(\mathbf{x}_O|\mathbf{x}_I) \tag{S7}$$

which is nothing but the entropy of the conditional distribution $p_{O|I}$, and whose expectation value over the input distribution is exactly the conditional entropy. Thus, we have

$$I_{IO} = H_O - \left\langle h_{O|I} \right\rangle_I = H_O - \int d\mathbf{x}_I \, p_I(\mathbf{x}_I) h_{O|I}(\mathbf{x}_I) \tag{S8}$$

which, as we will see, will allow us to evaluate the mutual information directly from samples of the joint distribution.

## S3. SUPPLEMENTARY NOTE 3: DIRECT INPUT-OUTPUT CONNECTIONS

Within the hierarchical scheme highlighted above, we first consider the instructive example of a system with only two units before considering the full three-unit system of the main text: an input unit, $\mathbf{x}_I$, with $M_I$ nodes, and an output

unit, $\mathbf{x}_O$, with $M_O$ nodes. The adjacency tensor is given by

$$\hat{A} = \begin{pmatrix} \hat{A}_I & 0 \\ \hat{A}_{OI} & \hat{A}_O \end{pmatrix} \tag{S9}$$

where $\hat{A}_I$ and $\hat{A}_O$ are $M_I \times M_I$ and $M_O \times M_O$ matrices, respectively, and $\hat{A}_{OI}$ describes the connections from the input to the output unit. This model contains two timescales and any processing mechanism converting input into output is effectively taken into account by the nonlinear inter-unit activation functions, as presented above. In order to obtain analytical solutions for the joint probability $p_{IO}(\mathbf{x}_I, \mathbf{x}_O, t)$, we focus on the limiting case of a slow input, $\tau_I \gg \tau_O$. Indeed, the other limit, albeit interesting from a mathematical perspective, will not give rise to any information between the units, as proven in [7]. We also focus on the steady-state solution, so we will neglect time dependencies.

In this limit we can rescale time by the slowest timescale, i.e., $t \to t/\tau_I$, and seek a solution of the form

$$p_{IO}(\mathbf{x}_I, \mathbf{x}_O, t) = p^{(0)}_{IO}(\mathbf{x}_I, \mathbf{x}_O, t) + \frac{\tau_O}{\tau_I} p^{(1)}_{IO}(\mathbf{x}_I, \mathbf{x}_O, t) + \mathcal{O}\left(\left(\frac{\tau_O}{\tau_I}\right)^2\right)$$

where the superscript denotes the order of the expansion in the small parameter, $\tau_O/\tau_I \ll 1$. Up to the first order, i.e., with $\mathcal{O}(\tau_O/\tau_I)$ corrections, the Fokker-Planck equation becomes

$$\frac{\partial}{\partial t} p^{(0)}_{IO} = \frac{\tau_I}{\tau_O} \mathcal{L}_O p^{(0)}_{IO} + \mathcal{L}_I p^{(0)}_{IO} + \mathcal{L}_O p^{(1)}_{IO} + \mathcal{O}\left(\frac{\tau_O}{\tau_I}\right)$$

where we suppressed the dependencies of $p_{IO}$ for brevity. Proceeding order-by-order, as outlined in [8], we obtain a solution for $p^{(0)}_{IO} := p^{\mathrm{st}}_{IO}$ of the form

$$p^{\mathrm{st}}_{IO}(\mathbf{x}_I, \mathbf{x}_O) = p^{\mathrm{st}}_I(\mathbf{x}_I)\, p^{\mathrm{st}}_{O|I}(\mathbf{x}_O|\mathbf{x}_I) \tag{S10}$$

where the probabilities are the stationary solutions of the operators

$$\mathcal{L}_I(\mathbf{x}_I)\, p^{\mathrm{st}}_I(\mathbf{x}_I) = 0, \qquad \mathcal{L}_{O|I}(\mathbf{x}_I, \mathbf{x}_O)\, p^{\mathrm{st}}_{O|I}(\mathbf{x}_O|\mathbf{x}_I) = 0 \tag{S11}$$

with

$$\mathcal{L}_{O|I}(\mathbf{x}_I, \mathbf{x}_O) = \sum_{i=1}^{M_O} \frac{\partial}{\partial x^i_O} \left[ \sum_{j=1}^{M_O} A^{ij}_O \left( x^j_O - \sum_{k=1}^{M_O} g_{OI} \left(A^{-1}_O\right)^{jk} \phi_{OI}\left(A^{k,1}_{OI}, \dots, A^{k,M_I}_{OI}; \mathbf{x}_I\right)\right) + D^i_O \frac{\partial}{\partial x^i_O} \right]. \tag{S12}$$

Crucially, both these operators admit a stationary multivariate Gaussian distribution $\mathcal{N}(\mathbf{m}, \hat{\Sigma})$, where $\mathbf{m}$ is the mean and $\hat{\Sigma}$ the covariance matrix. Indeed, the input evolves independently of the output and, as such, it is governed by a Fokker-Planck equation without inter-unit interactions. On the other hand, the conditional probability of the output given the input, $p^{\mathrm{st}}_{O|I}$, evolves according to an operator where interactions between the units only depend on the value of $\mathbf{x}_I$, which has to be considered quenched. Specifically, we have that

$$p^{\mathrm{st}}_I(\mathbf{x}_I) = \mathcal{N}_I\left(\mathbf{0}, \hat{\Sigma}_I\right), \qquad p^{\mathrm{st}}_{O|I}(\mathbf{x}_O|\mathbf{x}_I) = \mathcal{N}_O\left(\mathbf{m}_{O|I}(\mathbf{x}_I), \hat{\Sigma}_O\right) \tag{S13}$$

where

$$m^i_{O|I}(\mathbf{x}_I) = g_{OI} \sum_{k=1}^{M_O} \left(A^{-1}_O\right)^{ik} \phi_{OI}\left(A^{k,1}_{OI}, \dots, A^{k,M_I}_{OI}; \mathbf{x}_I\right), \quad i = 1, \dots, M_O \tag{S14}$$

and the covariance matrices obey the Lyapunov equations $\hat{A}_I \hat{\Sigma}_I + \hat{\Sigma}_I \hat{A}^T_I = 2\hat{D}_I$, with $\hat{D}_I = \mathrm{diag}\left(D^1_I, \dots, D^{M_I}_I\right)$, and similarly for the output unit. The fundamental advantage of the factorization in Eq. (S10) is that both its components are Gaussian. We also note that the structure of the solution remains identical independently on the form of the nonlinearity, so it is valid for both Eq. (S2) and Eq. (S3).

Yet, since the mean of $p^{\mathrm{st}}_{O|I}$ explicitly depends on $\mathbf{x}_I$, the output distribution, $p^{\mathrm{st}}_O = \int d\mathbf{x}_I\, p^{\mathrm{st}}_{O|I}\, p^{\mathrm{st}}_I$, obtained by marginalizing over $\mathbf{x}_I$, will not be Gaussian due to the nonlinearity of $\phi_{OI}$. Therefore, the output entropy appearing in the mutual information in Eq. (S8) cannot be evaluated analytically. However, the second term is given by

$$h_{O|I}(\mathbf{x}_I) = \frac{1}{2}\left[M_O\left(1 + \log_2(2\pi)\right) + \log_2 \det \hat{\Sigma}_O\right] \equiv h_{O|I} \tag{S15}$$

which does not depend on $\mathbf{x}_I$. As a consequence, we just need to evaluate $H_O$ numerically. In particular, we can easily sample the joint distribution $p^{\rm st}_{IO}$, and thus obtain samples from the output that can be used to estimate its distribution and entropy. We employ both a classic Vasicek estimator [9] and the standard Kozachenko-Leonenko kNN estimator [10], as we focus on the case of one-dimensional outputs. Due to the curse of dimensionality, in higher dimensions more refined strategies would be needed [11]. With an estimate $\tilde{H}_O$ of the output entropy at hand, the mutual information between the output and input unit simply reads

$$I_{IO} = \tilde{H}_O - \frac{1}{2}\left[M_O\left(1+\log_2(2\pi)\right) + \log_2 \det \hat{\Sigma}_O\right] . \tag{S16}$$

Although the joint probability in Eq. (S10) is nonlinear, the factorization into Gaussian conditional probabilities allows for an efficient sampling scheme. Indeed, we can obtain a sample $\{\mathbf{x}_I, \mathbf{x}_O\}$ by sampling first the Gaussian input, and then the output from $p^{\rm st}_{O|I}$, whose mean depend on $\mathbf{x}_I$.

### A. Effect of the linear stability of the input

We now study how the linear stability of the input affects the two-unit system described in the previous section. The input is stable if the spectral radius of $\hat{A}_I$, i.e., the absolute value of its largest eigenvalue, is smaller than 1. We consider an input-output system whose intra-units connections are described by the random matrix

$$A_I^{ij} \sim \mathcal{N}\left(0, \frac{\sigma_I}{\sqrt{M_I}}\right) \tag{S17}$$

where the usual normalization of the standard deviation of the elements of $\hat{A}_I$ ensures a proper scaling of its spectrum with the input dimensionality [12]. Since the output is one-dimensional, we simply set $A_O^{11} = 1$. Similarly,

$$A_{OI}^{ij} \sim \mathcal{N}\left(0, \sigma_{OI}\right) \tag{S18}$$

and we fix $\sigma_{OI} = 1$ for simplicity. In Figure S1 we show that $\sigma_I$ has a crucial impact on the output probability and the mutual information between the input and the output. In particular, $p_O$ shows an emergent bistability as $\sigma_I$ increases (Figure S1a-b). Furthermore, as expected from previous works [13], $I_{IO}$ increases as $\sigma_I$ approaches the edge of linear stability $\sigma_I^c$ (Figure S1c). The increase is more marked for the case of the nonlinear integration case, suggesting that, as the input becomes more variable in time, an activation function with nonlinear integration is able to better track the input features.

## S4. SUPPLEMENTARY NOTE 4: INTERMEDIATE PROCESSING BETWEEN THE INPUT AND THE OUTPUT

Since it is known that timescales play a pivotal role in shaping information propagation [7], we will explore their interplay with nonlinear interactions by adding an intermediate processing unit to the system that relays information. Since processing can, in principle, act on a different timescale, this more general system is characterized by three units: an input unit $\mathbf{x}_I$, with $M_I$ nodes; a processing unit $\mathbf{x}_P$, with $M_P$ nodes; and an output unit $\mathbf{x}_O$, with $M_O$ nodes. The adjacency tensor is

$$\hat{A} = \begin{pmatrix} \hat{A}_I & 0 & 0 \\ \hat{A}_{PI} & \hat{A}_P & 0 \\ 0 & \hat{A}_{OP} & \hat{A}_O \end{pmatrix} \tag{S19}$$

so that the input is given to the processing unit before it is passed on to the output. To retain a clear interpretation of input and output signals, we also maintain the hierarchical structure of the model. As before, $\hat{A}_I$, $\hat{A}_P$ $\hat{A}_O$ are $M_I \times M_I$, $M_P \times M_P$ and $M_O \times M_O$ matrices, respectively. $\hat{A}_{PI}$ describes the connections from the input to the processing unit, and $\hat{A}_{OP}$ the connections from the processing to the output unit. In order to study this system exactly, following [7], we again consider the input to be the slowest dof at play - so that it can generate a non-zero mutual information - with timescale $\tau_I$. Then, we distinguish between two cases: slow processing, i.e., $\tau_I \gg \tau_P \gg \tau_O$, and fast processing, i.e., $\tau_I \gg \tau_O \gg \tau_P$. As in the previous section, we aim to compute the mutual information between the input and the output, which we take to be one-dimensional for numerical stability.

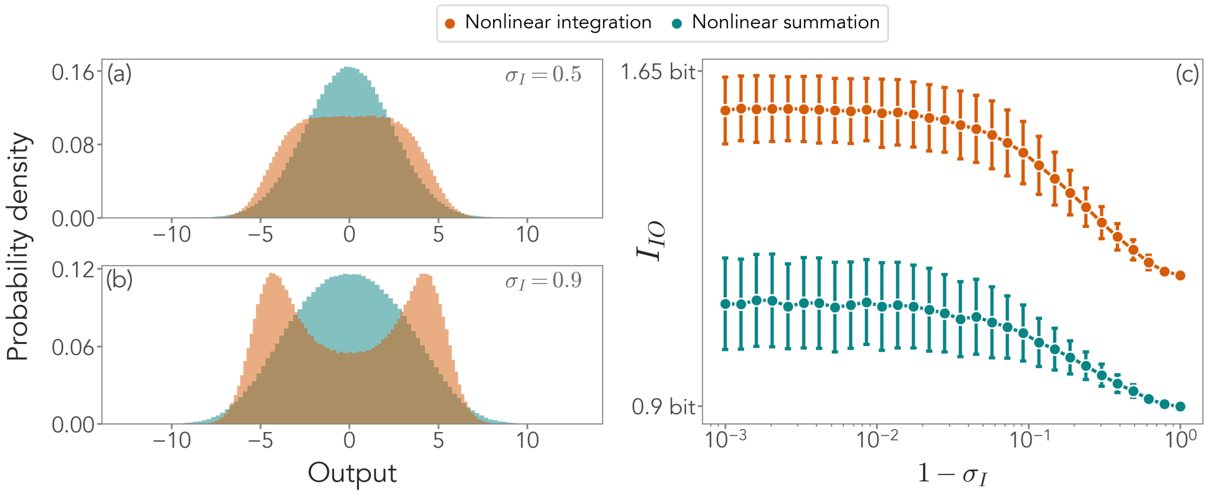


FIG. S1. Effect of the input stability in an input-output system, both with an activation function following a nonlinear summation and a nonlinear integration. In this figure, $M_I = 50$, $M_O = 1$, $g_{OI} = 5$, $\sigma_{OI} = 5$. (a-b) Output distributions for two different values of $\sigma_I$. As the input is closer to the edge of stability $\sigma_I^c = 1$, the distribution with nonlinear integration becomes bistable. (c) The mutual information between the input and the output, $I_{IO}$, increases as the input approaches the edge of linear stability, regardless of the type of activation function at hand. The increase is more marked for the nonlinear integration case.

### A. Fast processing

We proceed as in the two-unit case. After rescaling time as $t \to t/\tau_I$, we seek a solution of the form

$$p_{IPO}(\mathbf{x}_I, \mathbf{x}_P, \mathbf{x}_O, t) = p_{IPO}^{(P,0)}(\mathbf{x}_I, \mathbf{x}_P, \mathbf{x}_O, t) + \epsilon_P \left[ p_{IPO}^{(O,0)}(\mathbf{x}_I, \mathbf{x}_P, \mathbf{x}_O, t) + \epsilon_O p_{IPO}^{(I,1)}(\mathbf{x}_I, \mathbf{x}_P, \mathbf{x}_O, t) \right] + \mathcal{O}\left(\epsilon_P^2, \epsilon_O^2\right)$$

where $\epsilon_O = \tau_O/\tau_I \gg \epsilon_P = \tau_P/\tau_I$, and the superscript denotes the order in the corresponding term, distinguishing also between the variable it refers to, since there are as many zeroth order as the number of variables faster than the input. At leading order, the Fokker-Planck equation becomes

$$\frac{\partial}{\partial t} p_{IPO}^{(P,0)} = \left[ \mathcal{L}_I + \frac{\mathcal{L}_O}{\epsilon_O} + \frac{\mathcal{L}_P}{\epsilon_P} \right] p_{IPO}^{(P,0)} + \mathcal{L}_P p_{IPO}^{(O,0)}$$

which can be solved order-by-order. At order $\mathcal{O}(1/\epsilon_P)$, we find that

$$\mathcal{L}_P(\mathbf{x}_I, \mathbf{x}_P)\, p_{IPO}^{(P,0)}(\mathbf{x}_I, \mathbf{x}_P, \mathbf{x}_O, t) = 0$$

and, after a marginalization over the output state, $\mathbf{x}_O$, we have:

$$\mathcal{L}_P(\mathbf{x}_I, \mathbf{x}_P)\, p_{IP}^{(P,0)}(\mathbf{x}_I, \mathbf{x}_P, t) = 0 = p_I^{(P,0)}(\mathbf{x}_I, t) \mathcal{L}_P(\mathbf{x}_I, \mathbf{x}_P)\, p_{P|I}^{\rm st}(\mathbf{x}_P|\mathbf{x}_I) \implies \mathcal{L}_{P|I}(\mathbf{x}_I, \mathbf{x}_P)\, p_{P|I}^{\rm st}(\mathbf{x}_P|\mathbf{x}_I) = 0$$

where we introduced $\mathcal{L}_{P|I} := \mathcal{L}_P$, to emphasize that its stationary distribution $p_{P|I}^{\rm st}$ is evaluated at fixed input. Thus, a solution of the form $p_{IPO}^{(P,0)} = p_{P|I}^{\rm st} p_{IO}^{(P,0)}$ automatically solves the first order. In particular, as in the previous section, we have that

$$p_{P|I}^{\rm st}(\mathbf{x}_P|\mathbf{x}_I) = \mathcal{N}_P\left(\mathbf{m}_{P|I}(\mathbf{x}_I), \hat{\Sigma}_P\right) \tag{S20}$$

is a Gaussian distribution with a mean that depends nonlinearly on the input,

$$m_{P|I}^i(\mathbf{x}_I) = g_{PI} \sum_{k=1}^{M_P} \left(A_P^{-1}\right)^{ik} \phi_{PI}\left(A_{PI}^{k,1}, \ldots, A_{PI}^{k,M_I}; \mathbf{x}_I\right), \quad i = 1, \ldots, M_P, \tag{S21}$$

and a covariance obeying the Lyapunov equation $\hat{A}_P \hat{\Sigma}_P + \hat{\Sigma}_P \hat{A}_P^T = 2\hat{D}_P$.

At the next order, $\mathcal{O}(1/\epsilon_O)$, we have that

$$p^{\rm st}_{P|I}(\mathbf{x}_P|\mathbf{x}_I)\mathcal{L}_O(\mathbf{x}_P,\mathbf{x}_O)\,p^{(P,0)}_{IO}(\mathbf{x}_I,\mathbf{x}_O,t) = 0 = p^{\rm st}_{P|I}(\mathbf{x}_P|\mathbf{x}_I)p^{(P,0)}_I(\mathbf{x}_I,t)\mathcal{L}_O(\mathbf{x}_P,\mathbf{x}_O)\,p^{(P,0),\rm st}_{O|I}(\mathbf{x}_O|\mathbf{x}_I)\ .$$

To solve for $p^{(P,0),\rm st}_{O|I}$, due to the explicit dependence of $\mathcal{L}_O(\mathbf{x}_P,\mathbf{x}_O)$ on the processing state, we integrate over $\mathbf{x}_P$ to obtain the following effective Fokker-Planck operator

$$\mathcal{L}^{\rm eff}_O(\mathbf{x}_I,\mathbf{x}_O) := \int d\mathbf{x}_P\, p^{\rm st}_{P|I}(\mathbf{x}_P|\mathbf{x}_I)\mathcal{L}_O(\mathbf{x}_P,\mathbf{x}_O) \tag{S22}$$

leading to:

$$p^{(P,0)}_I(\mathbf{x}_I,t)\mathcal{L}^{\rm eff}_O(\mathbf{x}_I,\mathbf{x}_O)\,p^{(P,0),\rm st}_{O|I}(\mathbf{x}_O|\mathbf{x}_I) = 0\ .$$

Thus, if we introduce the effective stationary distribution

$$\mathcal{L}^{\rm eff}_O(\mathbf{x}_I,\mathbf{x}_O)p^{\rm eff,st}_{O|I}(\mathbf{x}_O|\mathbf{x}_I) = 0, \tag{S23}$$

we end up with $p^{(P,0)}_{IPO} = p^{\rm st}_{P|I}p^{\rm eff,st}_{O|I}p^{(P,0)}_I$. However, to find an explicit form for $p^{\rm eff,st}_{O|I}$, we need to find an analytic expression for the $\mathcal{L}^{\rm eff}_O$, and solve for its stationary state. By construction, we have:

$$\mathcal{L}^{\rm eff}_O(\mathbf{x}_I,\mathbf{x}_O) = \sum_{i=1}^{M_O}\frac{\partial}{\partial x^i_O}\left[\sum_{j=1}^{M_O}A^{ij}_O\left(x^j_O - g_{OP}\sum_{k=1}^{M_O}\left(A^{-1}_O\right)^{jk}\left\langle\phi^k_{OP}\right\rangle_P(\mathbf{x}_I)\right) + D^i_O\frac{\partial}{\partial x^i_O}\right]$$

with

$$\left\langle\phi^k_{OP}\right\rangle_P(\mathbf{x}_I) = \frac{1}{\sqrt{(2\pi)^{M_P}\det\hat\Sigma_P}}\int d\mathbf{x}_P\exp\left[-\frac{1}{2}(\mathbf{x}_P-\mathbf{m}_{P|I}(\mathbf{x}_I))^T\hat\Sigma_P^{-1}(\mathbf{x}_P-\mathbf{m}_{P|I}(\mathbf{x}_I))\right]\phi_{OP}\left(\mathbf{A}^k_{OP},\mathbf{x}_P\right)$$

where we introduced $\mathbf{A}^k_{OP} = (A^{k,1}_{OP},\ldots,A^{k,M_P}_{OP})$ for brevity.

### 1. *Nonlinear summation*

We now need to distinguish the two classes of nonlinear couplings in Eqs. (S2) and (S3), as the integral over $\mathbf{x}_P$ explicitly depends on the form employed for activation function implementing interactions between the units. We first consider the case of a nonlinear summation (indicating it by with the superscript ns), Eq. (S2), so that

$$\begin{aligned}\left\langle\phi^i_{OP}\right\rangle^{\rm ns}_P(\mathbf{x}_I) &\propto \int dx^1_P\ldots dx^j_P\ldots dx^{M_P}_P\exp\left[-\frac{1}{2}(\mathbf{x}_P-\mathbf{m}_{P|I}(\mathbf{x}_I))^T\hat\Sigma_P^{-1}(\mathbf{x}_P-\mathbf{m}_{P|I}(\mathbf{x}_I))\right]\sum_{j=1}^{M_P}\frac{A^{ij}_{OP}}{M_P}\tanh x^j_P\\ &\propto \sum_{j=1}^{M_P}\frac{A^{ij}_{OP}}{M_P}\int dx^j_P\exp\left[-\frac{1}{2\Sigma^{jj}_P}\left(x^j_P - m^j_{P|I}(\mathbf{x}_I)\right)^2\right]\tanh x^j_P\end{aligned}$$

where we neglected the normalization terms for brevity and exploited the fact that the hyperbolic tangent term only depends on the components of $\mathbf{x}_P$ separately, so that the marginalization of the Gaussian over the other $M_P-1$ components is straightforward and amounts to eliminating their corresponding rows and columns of the covariance matrix. However, finding an exact expression of the Gaussian average of the hyperbolic task is not trivial. To proceed further, we need first to rewrite the hyperbolic tangent as

$$\begin{aligned}\tanh z = \frac{\sinh z}{\cosh z} = \frac{1-e^{-2z}}{1+e^{-2z}} &= (1-e^{-2z})\sum_{n=0}^{\infty}(-1)^n e^{-2nz}\\ &= \sum_{n=0}^{\infty}(-1)^n\left[e^{-2nz}-e^{-2(n+1)z}\right] = \sum_{n=0}^{\infty}(-1)^n e^{-2nz} + \sum_{n=1}^{\infty}(-1)^n e^{-2nz}\\ &= 1 + 2\sum_{n=1}^{\infty}(-1)^n e^{-2nz}\end{aligned}$$

which is convergent if and only if $e^{-2z} < 1$, i.e., for $z > 0$. We now need to evaluate its Gaussian average, here also indicated as $\langle \tanh(z) \rangle_G$, namely

$$\begin{aligned}
\frac{1}{\sqrt{2\pi\sigma^2}} \int_{-\infty}^{+\infty} dz \tanh(z) e^{-\frac{(z-m)^2}{2\sigma^2}} &= \frac{1}{\sqrt{2\pi\sigma^2}} \left[ \int_0^{+\infty} dz \tanh(z) e^{-\frac{(z-m)^2}{2\sigma^2}} + \int_{-\infty}^{0} dz \tanh(z) e^{-\frac{(z-m)^2}{2\sigma^2}} \right] \\
&= \frac{1}{\sqrt{2\pi\sigma^2}} \left[ \int_0^{+\infty} dz \tanh(z) e^{-\frac{(z-m)^2}{2\sigma^2}} - \int_{0}^{+\infty} dz \tanh(z) e^{-\frac{(z+m)^2}{2\sigma^2}} \right] \\
&= \frac{1}{\sqrt{2\pi\sigma^2}} \left[ \int_0^{+\infty} dz \left( 1 + 2 \sum_{n=1}^{\infty} (-1)^n e^{-2nz} \right) e^{-\frac{(z-m)^2}{2\sigma^2}} - e^{-\frac{(z+m)^2}{2\sigma^2}} \right] \\
&= \operatorname{erf}\left( \frac{m}{\sqrt{2\sigma^2}} \right) + 2 \sum_{n=1}^{\infty} (-1)^n \int_0^{+\infty} \frac{dz}{\sqrt{2\pi\sigma^2}} e^{-2nz} \left[ e^{-\frac{(z-m)^2}{2\sigma^2}} - e^{-\frac{(z+m)^2}{2\sigma^2}} \right]
\end{aligned}$$

which is a convergent expression since the integral is evaluated for $z > 0$. Thus,

$$\begin{aligned}
\langle \tanh(z) \rangle_G &= \operatorname{erf}\left( \frac{m}{\sqrt{2\sigma^2}} \right) + \sum_{n=1}^{\infty} (-1)^n \left[ e^{2n(n\sigma^2 - m)} \left( \operatorname{erf}\left( \frac{m - 2n\sigma^2}{\sqrt{2\sigma^2}} \right) + 1 \right) - e^{2n(n\sigma^2 + m)} \left( \operatorname{erf}\left( \frac{-m - 2n\sigma^2}{\sqrt{2\sigma^2}} \right) + 1 \right) \right] \\
&= \operatorname{erf}\left( \frac{m}{\sqrt{2\sigma^2}} \right) + \sum_{n=1}^{\infty} (-1)^n e^{2n^2\sigma^2} \left[ e^{-2nm} \left( 1 + \operatorname{erf}\left( \frac{m - 2n\sigma^2}{\sqrt{2\sigma^2}} \right) \right) - e^{2nm} \operatorname{erfc}\left( \frac{m + 2n\sigma^2}{\sqrt{2\sigma^2}} \right) \right]
\end{aligned}$$

where $\operatorname{erfc}(z) = 1 - \operatorname{erf}(z)$. It is convenient to rewrite this expression as

$$\langle \tanh(z) \rangle_G = \operatorname{erf}\left( \frac{m}{\sqrt{2\sigma^2}} \right) + \sum_{n=1}^{\infty} (-1)^n e^{2n^2\sigma^2} \left[ V_n^+(m, \sigma^2) - V_n^-(m, \sigma^2) \right] \tag{S24}$$

where we introduced the functions

$$\begin{aligned}
V_n^+(m, \sigma^2) &= e^{-2nm} \operatorname{erfc}\left( \frac{2n\sigma^2 - m}{\sqrt{2\sigma^2}} \right) \\
V_n^-(m, \sigma^2) &= e^{2nm} \operatorname{erfc}\left( \frac{2n\sigma^2 + m}{\sqrt{2\sigma^2}} \right)
\end{aligned} . \tag{S25}$$

Eq. (S24) is an exact expression for the Gaussian average of the hyperbolic tangent. In practice, it can be efficiently evaluated numerically, as convergence with the number of terms in the sum over $n$ is typically quick, depending on the value of the mean and the variance, as shown in Figure S2.

Therefore, we immediately have that

$$\mathcal{L}_O^{\text{ns,eff}}(\mathbf{x}_I, \mathbf{x}_O) = \sum_{i=1}^{M_O} \frac{\partial}{\partial x_O^i} \left[ \sum_{j=1}^{M_O} A_O^{ij} \left( x_O^j - m_{O|I}^{\text{ns},j}(\mathbf{x}_I) \right) + D_O^i \frac{\partial}{\partial x_O^i} \right] \tag{S26}$$

where the mean is given by

$$\begin{aligned}
m_{O|I}^{\text{ns},i}(\mathbf{x}_I) = \frac{g_{OP}}{M_P} \sum_{j=1}^{M_O} \sum_{k=1}^{M_P} \left( A_O^{-1} \right)^{ij} A_{OP}^{jk} \Bigg[ \operatorname{erf}\left( \frac{m_{P|I}^k(\mathbf{x}_I)}{\sqrt{2\Sigma_P^{kk}}} \right) + \\
+ \sum_{n=1}^{\infty} (-1)^n e^{2n^2 \Sigma_P^{kk}} \left[ V_n^+ \left( m_{P|I}^k(\mathbf{x}_I), \Sigma_P^{kk} \right) - V_n^-(m_{P|I}^k(\mathbf{x}_I), \Sigma_P^{kk}) \right] \Bigg] .
\end{aligned} \tag{S27}$$

By comparison with the formulas of the main text, $\mathcal{F}(x^k, v^k)$ is equal to the term in the square brackets with $x^k \to m_{P|I}^k$ and $v^k \to \Sigma_P^{kk}$ in this case. The same will hold for all other similar expressions presented herein. We included the superscript NS also in the effective operator to emphasize the case here considered. Notice that the mean of the output inherits a direct dependence on the input $\mathbf{x}_I$ through the averaging procedure over the fast processing, a phenomenon typical of minimal propagation paths in timescale-separated multiscale systems. For a detailed discussion beyond the scope of this work, see [7].

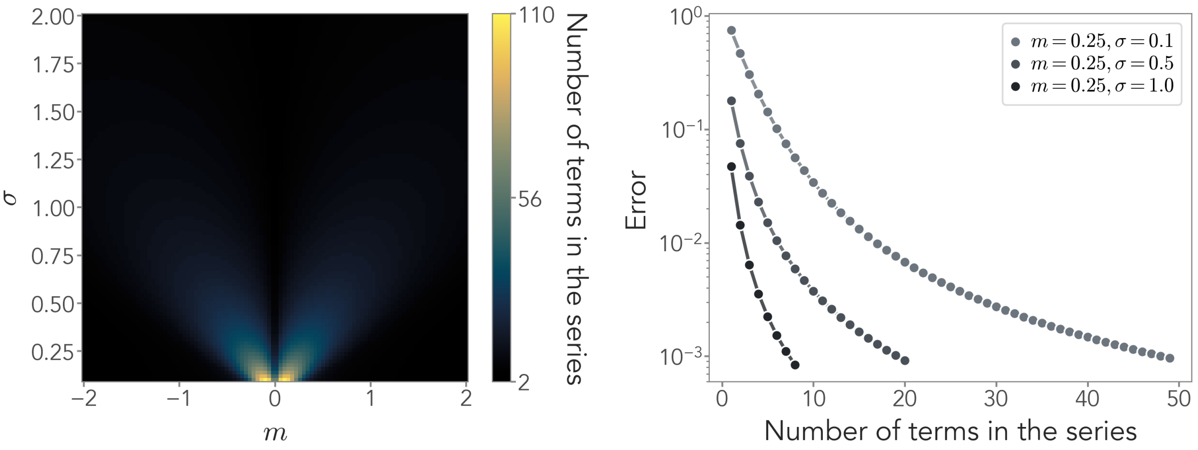


FIG. S2. Convergence of the series in Eq. (S24) with respect to the numerical integration of $\langle \tanh(z)\rangle_G$. The contour plot shows, for different mean and standard deviation, the number of terms in the series needed to reach an error of $10^{-3}$, i.e., an absolute difference between the truncated series and the numerical integral difference of $10^{-3}$.

### *2. Nonlinear integration*

We now switch to the case of nonlinear integration, Eq (S3). Contrary to the previous case, we cannot reduce the effective operator to a sum of independent one-dimensional integrals. Indeed, we have

$$\mathcal{L}_O^{\text{int,eff}}(\mathbf{x}_I, \mathbf{x}_O) = \sum_{i=1}^{M_O} \frac{\partial}{\partial x_O^i} \left[ \sum_{j=1}^{M_O} A_O^{ij} \left( x_O^j - g_{OP} \sum_{k=1}^{M_O} \left(A_O^{-1}\right)^{jk} \langle \phi_{OP}^k \rangle_P^{\text{int}} (\mathbf{x}_I) \right) + D_O^i \frac{\partial}{\partial x_O^i} \right]$$

where

$$\langle \phi_{OP}^i \rangle_P^{\text{int}} (\mathbf{x}_I) \propto \int dx_P^1 \dots dx_P^{M_P} \exp\left[ -\frac{1}{2} (\mathbf{x}_P - \mathbf{m}_{P|I}(\mathbf{x}_I))^T \hat{\Sigma}_P^{-1} (\mathbf{x}_P - \mathbf{m}_{P|I}(\mathbf{x}_I)) \right] \tanh\left( \sum_{j=1}^{M_P} \frac{A_{OP}^{ij}}{M_P} x_P^j \right) .$$

Here, we are using the superscript INT to highlight that these quantities only refer to the nonlinear integration case. In order to exploit the expansion of the hyperbolic tangent, we consider the change of variables $\mathbf{z} = \hat{C}_i \mathbf{x}_P$, where

$$\hat{C}_i = \begin{pmatrix} \mathbb{I}_{M_P - 1} & \mathbf{0} \\ \mathbf{A}_{OP}^i / M_P & \end{pmatrix} \tag{S28}$$

is a $M_P \times M_P$ matrix and $\mathbf{A}_{OP}^i = (A_{OP}^{i1}, \dots, A_{OP}^{iM_P})$. Thus, $\mathbf{z} = (z^1, \dots, z^{M_P}) = (x_P^1, \dots, x_P^{M_P - 1}, y_i)$ with $y_i = 1/M_P \sum_j A_{OP}^{ij} x_P^j$. After applying the change of variables, we end up with

$$\begin{aligned} \langle \phi_{OP}^i \rangle_P^{\text{int}} (\mathbf{x}_I) &\propto \int dz^1, \dots, dz^{M_P - 1} dy_i \exp\left[ -\frac{1}{2} (\mathbf{z} - \hat{C}_i \mathbf{m}_{P|I}(\mathbf{x}_I))^T (\hat{C}_i \hat{\Sigma}_P \hat{C}_i^T)^{-1} (\mathbf{z} - \hat{C}_i \mathbf{m}_{P|I}(\mathbf{x}_I)) \right] \tanh y_i \\ &\propto \int_{-\infty}^{+\infty} dy_i \exp\left[ -\frac{1}{2 v_{\text{int}}^i} \left( y_i - \frac{1}{M_P} \sum_{j=1}^{M_P} A_{OP}^{ij} m_{P|I}^j (\mathbf{x}_I) \right)^2 \right] \tanh y_i \end{aligned}$$

after the integration over $z^1, \dots, z^{M_P - 1}$. Here, we introduced the variance of the resulting marginal distribution

$$v_{\text{int}}^i = \sum_{j=1}^{M_P} \sum_{k=1}^{M_P} C_i^{M_P j} \Sigma_P^{jk} C_i^{M_P k} = \frac{1}{M_P^2} \sum_{j=1}^{M_P} \sum_{k=1}^{M_P} A_{OP}^{ij} A_{OP}^{ik} \Sigma_P^{jk} \tag{S29}$$

and mean

$$m_{\text{int}}^i (\mathbf{x}_I) = \frac{1}{M_P} \sum_{j=1}^{M_P} A_{OP}^{ij} m_{P|I}^j (\mathbf{x}_I) . \tag{S30}$$

Therefore, we can now proceed as in the previous case, so that

$$\langle \phi_{OP}^i \rangle_P^{\text{int}} (\mathbf{x}_I) = \text{erf}\left(\frac{m_{\text{int}}^i(\mathbf{x}_I)}{\sqrt{2 v_{\text{int}}^i}}\right) + \sum_{n=1}^{\infty} (-1)^n e^{2n^2 v_{\text{int}}^i} \left[V_n^+(m_{\text{int}}^i(\mathbf{x}_I), v_{\text{int}}^i) - V_n^-(m_{\text{int}}^i(\mathbf{x}_I), v_{\text{int}}^i)\right]$$

and the effective output operator now reads

$$\mathcal{L}_O^{\text{int,eff}}(\mathbf{x}_I, \mathbf{x}_O) = \sum_{i=1}^{M_O} \frac{\partial}{\partial x_O^i} \left[\sum_{j=1}^{M_O} A_O^{ij} \left(x_O^j - m_{O|I}^{\text{int},j}(\mathbf{x}_I)\right) + D_O^i \frac{\partial}{\partial x_O^i}\right] \tag{S31}$$

where the mean is given by

$$m_{O|I}^{\text{int},i}(\mathbf{x}_I) = g_{OP} \sum_{j=1}^{M_O} \left(A_O^{-1}\right)^{ij} \left[\text{erf}\left(\frac{m_{\text{int}}^j(\mathbf{x}_I)}{\sqrt{2 v_{\text{int}}^j}}\right) + \right.$$
$$\left. + \sum_{n=1}^{\infty} (-1)^n e^{2n^2 v_{\text{int}}^j} \left[V_n^+\left(m_{\text{int}}^j(\mathbf{x}_I), v_{\text{int}}^j\right) - V_n^-(m_{\text{int}}^j(\mathbf{x}_I), v_{\text{int}}^j)\right]\right] . \tag{S32}$$

Notice that, as before, the mean of the output inherits a direct dependence on the input $\mathbf{x}_I$ through the averaging over the fast processing. However, contrary to the case of a nonlinear sum, the integration term makes the dependence on the inter-unit interactions highly nonlinear, as they appear in both $\mathbf{m}_{\text{int}}$ and $\mathbf{v}_{\text{int}}$.

### 3. *Computing the mutual information*

In both scenarios, we have an exact expression for $p_{O|I}^{\text{eff,st}}$ which is a Gaussian distribution in the output state $\mathbf{x}_O$ with a nonlinear dependence on the input state $\mathbf{x}_I$. Therefore, we only need to solve the last order of the Fokker-Planck equation, which now reads

$$p_{P|I}^{\text{st}} p_{O|I}^{\text{eff,st}} \frac{\partial}{\partial t} p_{IPO}^{(P,0)} p_I^{(P,0)} = p_{P|I}^{\text{st}} p_{O|I}^{\text{eff,st}} \mathcal{L}_I p_I^{(P,0)} + \mathcal{L}_P p_{IPO}^{(O,0)}$$

at order $\mathcal{O}(1)$. By integrating over $\mathbf{x}_P$ and ignoring the vanishing border term, we end up with

$$\frac{\partial}{\partial t} p_I^{(P,0)} = \mathcal{L}_I p_I^{(P,0)} \tag{S33}$$

which immediately leads to the stationary Gaussian distribution $p_I^{(P,0)} := p_I^{\text{st}} = \mathcal{N}_I(0, \hat{\Sigma}_I)$, with $\hat{A}_I \hat{\Sigma}_I + \hat{\Sigma}_I \hat{A}_I^T = 2\hat{D}_I$. This is expected, as the input evolves independently on the other degrees of freedom. Overall, we can write the leading-order solution of the joint probability distribution as follows:

$$p_{IPO}^{(P,0)} := p_{IPO}^{\text{fp}} = p_{P|I}^{\text{st}} \left(\mathbf{m}_{P|I}(\mathbf{x}_I)\right) p_{O|I}^{\text{eff,st}} \left(\mathbf{m}_{O|I}(\mathbf{x}_I)\right) p_I^{\text{st}} \tag{S34}$$

where the superscript stands for "fast processing" and we highlighted that the dependencies of the conditional distributions enter through their means. As in the case of two units, $p_{IPO}^{\text{fp}}$ is a highly nonlinear distribution. However, our factorization into Gaussian distributions allows for its efficient sampling, as all the nonlinearities appear in the mean as conditional dependencies. In particular, we can compute the mutual information between the input and the output. We immediately have that their joint distribution is

$$p_{IO}^{\text{fp}} = p_{O|I}^{\text{eff,st}} p_I^{\text{st}}$$

so that

$$h_{O|I}(\mathbf{x}_I) = \frac{1}{2}\left[M_O\left(1 + \log_2(2\pi)\right) + \log_2 \det \hat{\Sigma}_O\right] \equiv h_{O|I} \tag{S35}$$

since the nonlinear dependencies of $p_{O|I}^{\text{eff,st}}$ on $\mathbf{x}_I$ do not appear in its covariance matrix. Thus, we only need to evaluate $H_O$ numerically to estimate $I_{IO} = H_O - h_{O|I}$. We can proceed as detailed in Section S3. We can easily sample the joint distribution $p_{IO}^{\text{fp}}$ by leveraging its Gaussian factorization:

1. sample $\{\mathbf{x}_I\}_{i=1}^{N_{\rm sam}}$ from the independent Gaussian distribution of the input;
2. compute the means $\mathbf{m}_{O|I}(\{\mathbf{x}_I\}_i)$ through either Eq. (S27) or Eq. (S32), depending on the nonlinearity, for each sample $i$;
3. for all $i$, sample $\mathbf{x}_O$ from the multivariate Gaussian with covariance $\hat\Sigma_O$ and means $\mathbf{m}_{O|I}(\{\mathbf{x}_I\}_i)$.

Then, the entropy $H_O$ of the output distribution can be estimated from the samples $\{\mathbf{x}_O\}_i$ [9, 10]. Since we focus on one-dimensional outputs, such estimates are especially robust as they do not suffer from the curse of dimensionality.

### B. Slow processing

We now focus on the case $\tau_I \gg \tau_P \gg \tau_O$, i.e., of a processing unit that is much slower than the output. As before, after rescaling time by the slowest timescale $t \to t/\tau_I$, we seek a solution of the form

$$p_{IPO}(\mathbf{x}_I, \mathbf{x}_P, \mathbf{x}_O, t) = p^{(O,0)}_{IPO}(\mathbf{x}_I, \mathbf{x}_P, \mathbf{x}_O, t) + \epsilon_O \left[p^{(P,0)}_{IPO}(\mathbf{x}_I, \mathbf{x}_P, \mathbf{x}_O, t) + \epsilon_P p^{(I,1)}_{IPO}(\mathbf{x}_I, \mathbf{x}_P, \mathbf{x}_O, t)\right] + \mathcal{O}\left(\epsilon_O^2, \epsilon_P^2\right)$$

where now $\epsilon_O = \tau_O/\tau_I \ll \epsilon_P = \tau_P/\tau_I$. At leading order, the Fokker-Planck equation becomes

$$\frac{\partial}{\partial t} p^{(O,0)}_{IPO} = \left[\mathcal{L}_I + \frac{\mathcal{L}_O}{\epsilon_O} + \frac{\mathcal{L}_P}{\epsilon_P}\right] p^{(O,0)}_{IPO} + \mathcal{L}_P p^{(P,0)}_{IPO}$$

and, at order $\mathcal{O}(1/\epsilon_O)$, we find that

$$\mathcal{L}_O(\mathbf{x}_P, \mathbf{x}_O)\, p^{(O,0)}_{IPO}(\mathbf{x}_I, \mathbf{x}_P, \mathbf{x}_O, t) = 0\ .$$

After a marginalization over the input state, $\mathbf{x}_I$, we have:

$$\mathcal{L}_O(\mathbf{x}_P, \mathbf{x}_O)\, p^{(O,0)}_{PO}(\mathbf{x}_P, \mathbf{x}_O, t) = 0 = p^{(O,0)}_P(\mathbf{x}_P, t)\mathcal{L}_O(\mathbf{x}_P, \mathbf{x}_O)\, p^{\rm st}_{O|P}(\mathbf{x}_O|\mathbf{x}_P) \implies \mathcal{L}_{O|P}(\mathbf{x}_P, \mathbf{x}_O)\, p^{\rm st}_{O|P}(\mathbf{x}_O|\mathbf{x}_P) = 0$$

where for notational clarity $\mathcal{L}_{O|P} := \mathcal{L}_O$, denoting that its stationary distribution $p^{\rm st}_{O|P}$ is obtained at a fixed processing state. Thus, a solution of the form $p^{(P,0)}_{IPO} = p^{\rm st}_{O|P} p^{(O,0)}_{IP}$ automatically solves the leading order considered above. In particular, as in the previous section, we have that

$$p^{\rm st}_{O|P}(\mathbf{x}_O|\mathbf{x}_P) = \mathcal{N}_O\left(\mathbf{m}_{O|P}(\mathbf{x}_P), \hat\Sigma_O\right) \tag{S36}$$

is a Gaussian distribution with a mean that depends nonlinearly on the processing state,

$$m^i_{O|P}(\mathbf{x}_P) = g_{OP} \sum_{k=1}^{M_O} \left(A_O^{-1}\right)^{ik} \phi_{OP}\left(A^{k,1}_{OP}, \dots, A^{k,M_P}_{OP}; \mathbf{x}_P\right), \quad i = 1, \dots, M_O, \tag{S37}$$

and a covariance obeying the Lyapunov equation $\hat A_O \hat\Sigma_O + \hat\Sigma_O \hat A_O^T = 2\hat D_O$.

At the next order, $\mathcal{O}(1/\epsilon_P)$, we find

$$p^{\rm st}_{O|P}(\mathbf{x}_O|\mathbf{x}_P)\mathcal{L}_P(\mathbf{x}_I, \mathbf{x}_P)\, p^{(O,0)}_{IP}(\mathbf{x}_I, \mathbf{x}_P, t) = 0 = p^{\rm st}_{O|P}(\mathbf{x}_O|\mathbf{x}_P) p^{(O,0)}_I(\mathbf{x}_I, t)\mathcal{L}_P(\mathbf{x}_I, \mathbf{x}_P)\, p^{(O,0),\rm st}_{P|I}(\mathbf{x}_P|\mathbf{x}_I)\ .$$

Contrarily to the case of the previous section, the integration over $\mathbf{x}_O$ can now be carried out immediately, since the operator does not depend on the output state, leading to

$$\mathcal{L}_{P|I}(\mathbf{x}_I, \mathbf{x}_P)\, p^{\rm st}_{P|I}(\mathbf{x}_P|\mathbf{x}_I) = 0 \tag{S38}$$

where, as before, we introduced $\mathcal{L}_{P|I} := \mathcal{L}_P$ to denote that its stationary distribution $p^{\rm st}_{P|I} = p^{(O,0),\rm st}_{P|I}(\mathbf{x}_P|\mathbf{x}_I)$ is obtained at a fixed input state. In particular, this is once more a Gaussian distribution where the nonlinear dependencies enter in the form of conditional dependencies of the mean on $\mathbf{x}_I$:

$$p^{\rm st}_{P|I}(\mathbf{x}_P|\mathbf{x}_I) = \mathcal{N}_P\left(\mathbf{m}_{P|I}(\mathbf{x}_I), \hat\Sigma_P\right) \tag{S39}$$

where

$$m^i_{P|I}(\mathbf{x}_I) = g_{PI} \sum_{k=1}^{M_P} \left(A_P^{-1}\right)^{ik} \phi_{PI}\left(A_{PI}^{k,1}, \ldots, A_{PI}^{k,M_I}; \mathbf{x}_I\right), \quad i = 1, \ldots, M_P, \tag{S40}$$

and the covariance matrix solves $\hat{A}_P \hat{\Sigma}_P + \hat{\Sigma}_P \hat{A}_P^T = 2\hat{D}_P$. Thus, we end up with $p_{IPO}^{(O,0)} = p^{\rm st}_{O|P} p^{\rm st}_{P|I} p_I^{(O,0)}$.

At order $\mathcal{O}(1)$, finally, we simply have that

$$\frac{\partial}{\partial t} p_I^{(O,0)} = \mathcal{L}_I p_I^{(O,0)} \implies p_I^{(O,0)} := p_I^{\rm st} = \mathcal{N}_I(0, \hat{\Sigma}_I) \tag{S41}$$

with $\hat{A}_I \hat{\Sigma}_I + \hat{\Sigma}_I \hat{A}_I^T = 2\hat{D}_I$. Once more, this highlights that the input evolves independently on the other degrees of freedom. Overall, we find that the Fokker-Planck equation is solved at leading order by

$$p_{IPO}^{(O,0)} := p_{IPO}^{\rm sp} = p^{\rm st}_{O|P}\left(\mathbf{m}_{O|P}(\mathbf{x}_P)\right) p^{\rm st}_{P|I}\left(\mathbf{m}_{P|I}(\mathbf{x}_I)\right) p_I^{\rm st} \tag{S42}$$

where the superscript stands for "slow processing" and we highlighted that the dependencies of the conditional distributions enter through their means. This expression is formally identical for both nonlinear scenarios, even if they change the internal functional dependencies and, as such, the overall shape of the distribution. Once again, $p_{IPO}^{\rm sp}$ is a highly nonlinear distribution, but the way the conditional dependencies appear is crucially different than the case of a fast processing unit and only depends on the timescale ordering considered. This is particularly relevant for the mutual information between the input and the output, since the distribution

$$p_{IO}^{\rm sp}(\mathbf{x}_I, \mathbf{x}_O) = p_I^{\rm st}(\mathbf{x}_I) p^{\rm st}_{O|I}(\mathbf{x}_O|\mathbf{x}_I) = p_I^{\rm st}(\mathbf{x}_I) \int d\mathbf{x}_P p^{\rm st}_{O|P}(\mathbf{x}_O|\mathbf{x}_P) p^{\rm st}_{P|I}(\mathbf{x}_P|\mathbf{x}_I) \tag{S43}$$

cannot be easily computed. Thus, the entropy of the conditional distribution $h_{O|I}(\mathbf{x}_I)$ is not known analytically.

To address this issue, we exploit the fact that we can efficiently sample $p^{\rm st}_{O|I}$, allowing us to easily obtain a numerical estimate of $h_{O|I}(\mathbf{x}_I)$. Then, we can estimate the conditional entropy

$$H_{O|I} = \int d\mathbf{x}_I p_I^{\rm st}(\mathbf{x}_I) h_{O|I}(\mathbf{x}_I) \tag{S44}$$

with importance sampling. We proceed as follows:

1. sample a fixed input $\mathbf{x}_I^{(i)} \sim \mathcal{N}_I(0, \hat{\Sigma}_I)$ for $i = 1, \ldots, N_{{\rm sam},I}$;

2. for each input sample $\mathbf{x}_I^{(i)}$, compute $\mathbf{m}_{P|I}\left(\mathbf{x}_I^{(i)}\right)$, and extract the samples $\mathbf{x}_P^{(i,j)}$ from $\mathcal{N}_P\left(\mathbf{m}_{P|I}\left(\mathbf{x}_I^{(i)}\right), \hat{\Sigma}_P\right)$ for $j = 1, \ldots, N_{\rm sam}$;

3. for each processing sample $\mathbf{x}_P^{(i,j)}$, compute the mean $\mathbf{m}_{O|P}\left(\mathbf{x}_P^{(i,j)}\right)$ and extract the corresponding output $\mathbf{x}_O^{(i,j)}$ from $\mathcal{N}_O\left(\mathbf{m}_{O|P}\left(\mathbf{x}_P^{(i,j)}\right), \hat{\Sigma}_O\right)$;

4. for each input sample $\mathbf{x}_I^{(i)}$, estimate the entropy $h_{O|I}\left(\mathbf{x}_I^{(i)}\right)$ of the conditional distribution $p^{\rm st}_{O|I}$ from the output samples $\{\mathbf{x}_O\}_{i,j}$, using any numerical estimator (e.g., Vasicek [9] or Kozachenko-Leonenko [10]);

5. estimate the conditional entropy $H_{O|I}$ via importance sampling,

$$H_{O|I} \approx \sum_{i=1}^{N_{{\rm sam},I}} h_{O|I}\left(\mathbf{x}_I^{(i)}\right) \tag{S45}$$

6. from all the output samples $\{\mathbf{x}_O\}_{i,j}$, estimate the entropy $H_O$ via any numerical estimator;

7. compute the mutual information as $I_{IO} = H_O - H_{O|I}$.

Once more, since we focus on one-dimensional outputs, this sampling scheme avoids any issue with the curse of dimensionality, allowing us to explore large processing (and input) dimensions.

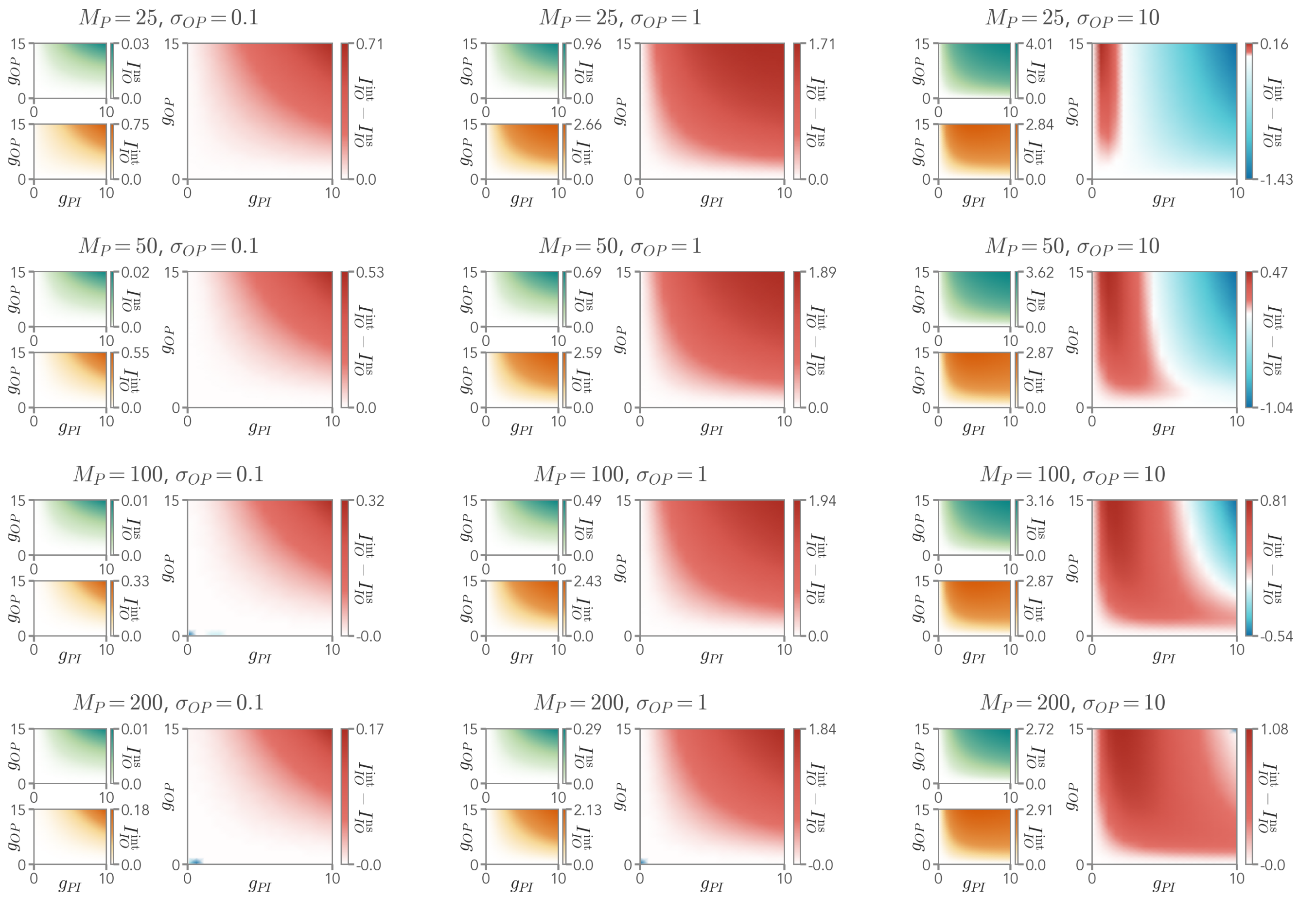


FIG. S3. Mutual information between the input and the output in the fast processing case, both for an activation function implementing a nonlinear summation ($I_{IO}^{\rm ns}$, teal) and a nonlinear integration ($I_{IO}^{\rm int}$, orange), for different size of the processing unit ($M_P$) and with different standard deviations $\sigma_{OP}$ of the interaction matrix $\hat{A}_{OP} \sim \mathcal{N}(0, \sigma_{OP})$. In this Figure, $M_I = 50$, $\sigma_I = \sigma_P = 0.9$, and $\sigma_{PI} = 1$. At large variances and in strong coupling regimes, a high-dimensional processing unit is needed for nonlinear integration to provide more information than nonlinear summation. This is due to the fact that, if $M_p$ is small and $\sigma_{OP}$ is large, the entries of the interaction matrix $\hat{A}_{OP}$ will be very different, pushing the activation function in the saturation regime. All information is measured in bits. Results are averaged over $10^3$ realization of the random matrices. For each realization, $N_{\rm sam} = 10^4$.

### C. Effect of the dimensionality of the processing

We now briefly study the interplay between the dimensionality of the processing unit, $M_P$, and the couplings between the units. In particular, as in the main text, we take the internal couplings of the units to be described by the random matrices

$$A_I^{ij} \sim \mathcal{N}\left(0, \frac{\sigma_I}{\sqrt{M_I}}\right), \quad A_P^{ij} \sim \mathcal{N}\left(0, \frac{\sigma_I}{\sqrt{M_I}}\right), \quad A_O = 1 \tag{S46}$$

where the output is one-dimensional, and the usual normalization of the standard deviations of the elements of $\hat{A}_I$ and $\hat{A}_P$ ensures a proper scaling of their spectrum with the dimensionality of the units. Similarly, the interactions between the units are given by the random matrices

$$A_{PI}^{ij} \sim \mathcal{N}\left(0, \sigma_{PI}\right), \quad A_{OP}^{ij} \sim \mathcal{N}\left(0, \sigma_{OP}\right) \tag{S47}$$

and we fix $\sigma_{OP} = 1$ for simplicity.

We first consider the case of a fast processing unit. In Figure S3, we show how the mutual information between the input and the output behaves as a function of the couplings $g_{PI}$ and $g_{OP}$ and at different values of $M_P$ and $\sigma_{OP}$, for

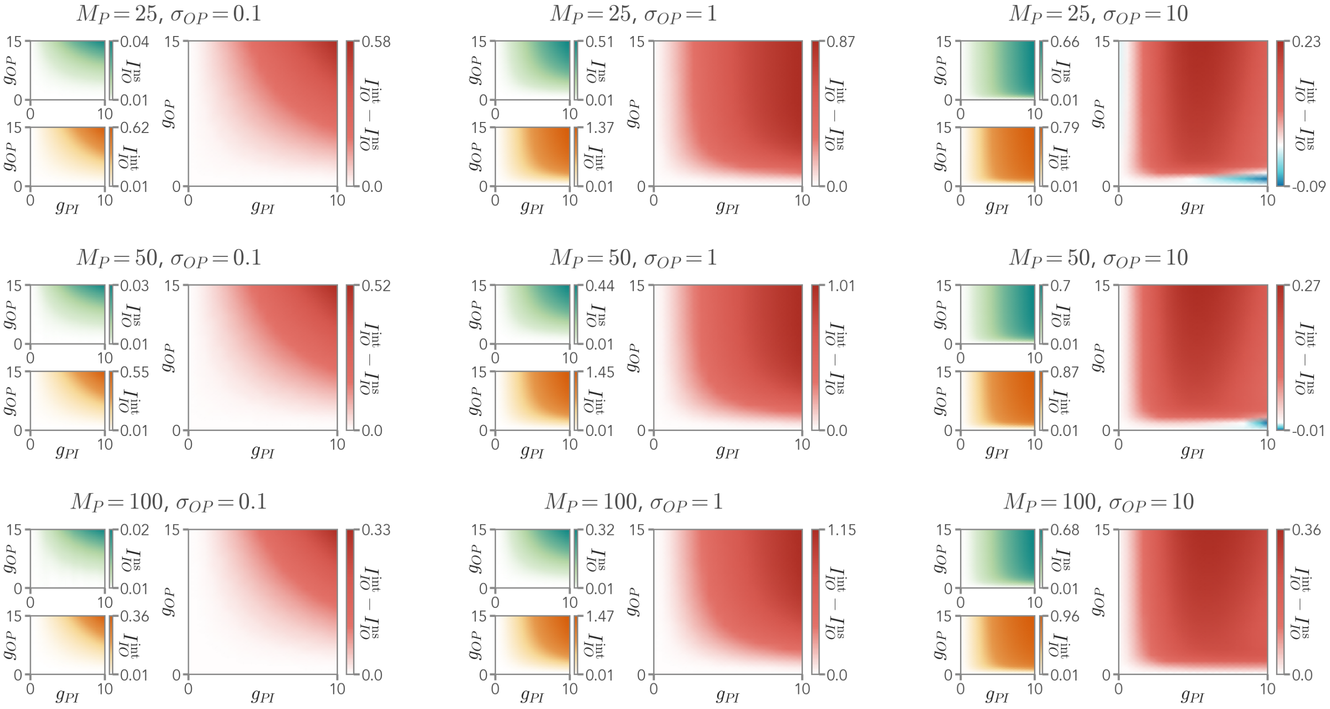


FIG. S4. Mutual information between the input and the output in the slow processing case, both for an activation function implementing a nonlinear summation ($I_{IO}^{\mathrm{ns}}$, teal) and a nonlinear integration ($I_{IO}^{\mathrm{int}}$, orange), for different size of the processing unit ($M_P$) and with different standard deviations $\sigma_{OP}$ of the interaction matrix $\hat{A}_{OP} \sim \mathcal{N}(0, \sigma_{OP})$. In this Figure, $M_I = 50$, $\sigma_I = \sigma_P = 0.9$, and $\sigma_{PI} = 1$. In this slow-processing case, information is typically smaller than in the fast-processing one. However, information is larger with nonlinear integration rather than nonlinear summation even for large variances $\sigma_{OP}^2$ and small processing dimensions $M_P$. All information is measured in bits. Results are averaged over $10^3$ realization of the random matrices. For each realization, $N_{\mathrm{sam},I} = 2 \cdot 10^3$ and $N_{\mathrm{sam}} = 10^3$.

an activation function implementing both a nonlinear summation and a nonlinear integration. Remarkably, we find that the dimensionality of the processing unit has a relevant effect at large interaction variances $\sigma_{OP}^2$. In particular, if $M_P$ is small enough and in strong coupling regimes, we find that the nonlinear summation may provide a larger mutual information than the nonlinear integration. Intuitively, this happens due to the highly nonlinear dependencies of the mean $\mathbf{m}_{O|I}$ (Eqs. (S27) and (S32)) on interaction matrix $\hat{A}_{OP}$ and the variance of the specific realization of its elements. As $M_P$ increases, the elements of $\hat{A}_{OP}$ are more uniformly sampled from the underlying Gaussian distribution, and this effect becomes less and less prominent and eventually disappears. Furthermore, we find that at small variances ($\sigma_{OP} = 0.1$) the mutual information $I_{IO}$ tends to be vanishingly small for a nonlinear summation, whereas it is significantly larger in the nonlinear integration case, especially at low processing dimensionalities.

Then, we switch to the case of a slow processing unit in Figure S4. Remarkably, the dimensionality of the processing is less impactful. In particular, in the coupling regimes we explored, the region where $I_{IO}^{\mathrm{ns}} > I_{IO}^{\mathrm{int}}$ is smaller and becomes negligible already at $M_P = 50$. Furthermore, we find that the input-output mutual information is consistently smaller with respect to the fast-processing case, as in the main text, suggesting that the timescales of the different units play a quantitative role in determining the information-processing capabilities of the system.

Finally, in Figure S5 we investigate more in detail the behavior of the mutual information with a fast processing unit and nonlinear integration at different $M_I$ and $M_P$. As reported in the main text, we find an optimal value of $M_P = M_P^*$ that maximizes $I_{IO}^{\mathrm{int}}$ for a given $M_I$. The optimal processing size increases as the input sizes become smaller, and this effect becomes prominent at sufficiently strong coupling regimes - either large $g_{OP}$ or large $\sigma_{OP}$. In Figure S7 we systematically compare the whole $(M_P, M_I)$ space for a specific choice of the couplings, $g_{OP} = g_{PI} = 10$, both for nonlinear integration and nonlinear summation. We show how $I_{IO}$ changes with $M_I$ and $M_P$ for a fast processing unit. We find that information typically decreases with $M_P$, regardless of the input dimension, when the activation function implements a nonlinear summation. This picture is markedly different than that of nonlinear integration, where at small input dimensions the mutual information between the input and the output is higher for large processing dimensionalities, highlighting once more the information-theoretic differences between the two schemes.

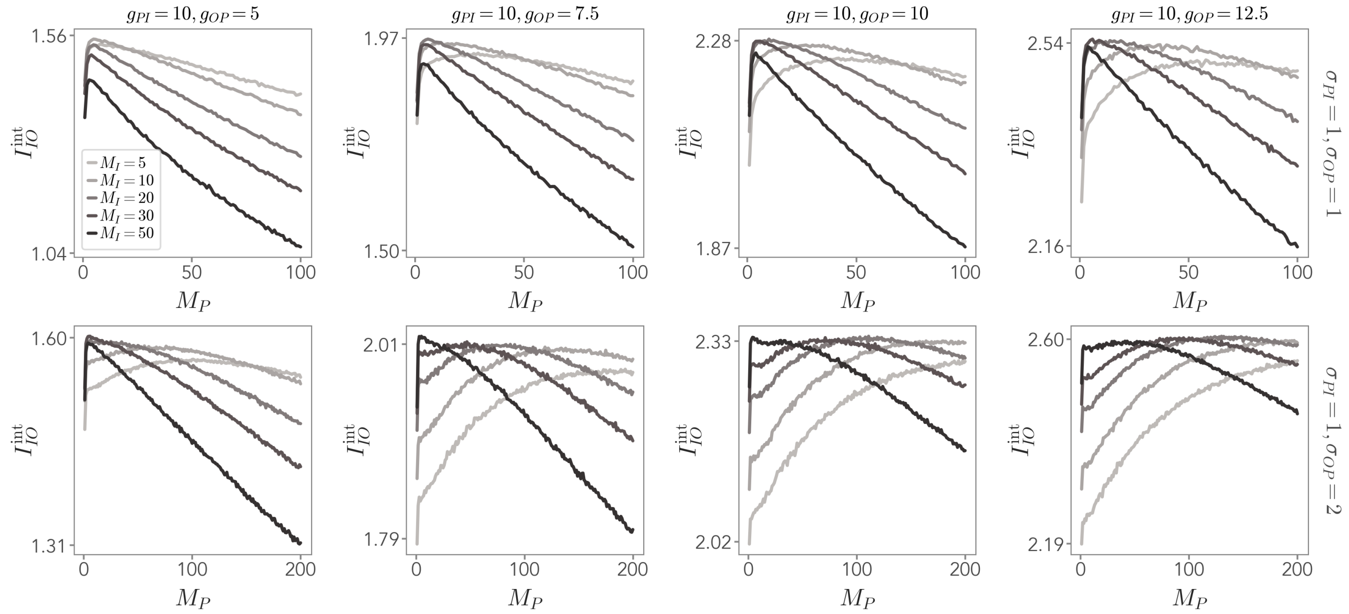


FIG. S5. Mutual information between the input and the output in the fast processing case, for an activation function implementing nonlinear integration. We compare $I_{IO}^{\text{int}}$ for different input and processing dimensions, respectively $M_I$ and $M_P$. In a strong coupling regime - defined by either large $g_{OP}$ or large $\sigma_{OP}$ - there exists an optimal processing dimensionality $M_P^*$ for a given input dimension $M_I$ that maximizes the input-output information. For $\sigma_{OP}=1$, this effect is particularly relevant at large $g_{OP}$, whereas it is more widespread for $\sigma_{OP}=2$. All information is measured in bits. In this Figure, $\sigma_I=\sigma_P=0.9$. Results are obtained by averaging over $2\cdot 10^4$ realization of the random interaction matrices.

### D. Effect of the topology of the processing and sparsity of connections

In Figure S6, we show how the topology of the processing unit and the sparsity of inter-unit connections affect the input-output mutual information. These two ingredients play different roles in determining the processing performance of the system. Indeed, as already commented in the main text, by changing the topology of the processing unit, we are not modifying its effective dimensionality, $M_P$, but only its internal distribution. The resulting effect is that sparser processing networks favor nonlinear integration, an observation that appears to be robust across various paradigmatic topologies. In Figure S6, we complement the analysis presented in the main text by studying Erdos-Renyi and small-world networks with different parameters. Our results suggest that a higher degree of the processing topology typically favors nonlinear summation, whereas integration performs better with sparser processing units.

Conversely, a reduction in the connection between layers results in a lower effective dimensionality that, as we already highlighted in the main text, favors nonlinear summation. In Figure S6, we consider different cases for the probability of connection between the nodes of two units, $p_{\text{unit}}$. In particular, we take $p_{\text{unit}}$ to be both fixed for all nodes ($p_{\text{unit}}=0.5$) or to be randomly extracted from a uniform distribution for each node ($p_{\text{unit}}\in[0,0.5]$, $p_{\text{unit}}\in[0,1]$, and $p_{\text{unit}}\in[0.5,1]$). We find that only the average number of connections between layers is important to qualitatively determine which nonlinear operations lead to higher mutual information. These results hint at the fact that a small number of global parameters might be relevant to capture the processing performance of a complex nonlinear system, such as the degree of the processing unit or the average number of connections between the units.

## S5. SUPPLEMENTARY NOTE 5: EMERGENT OUTPUT BISTABILITY

As shown in the main text, the output distribution may be bimodal, depending on the choice of the processing parameters. We measure this bistability of the underlying Langevin dynamics $x_O(t)$ by computing Sarle's bimodality coefficient [14], defined as:

$$b=\frac{s^2+1}{\kappa+q(n_{\text{samples}})} \tag{S48}$$

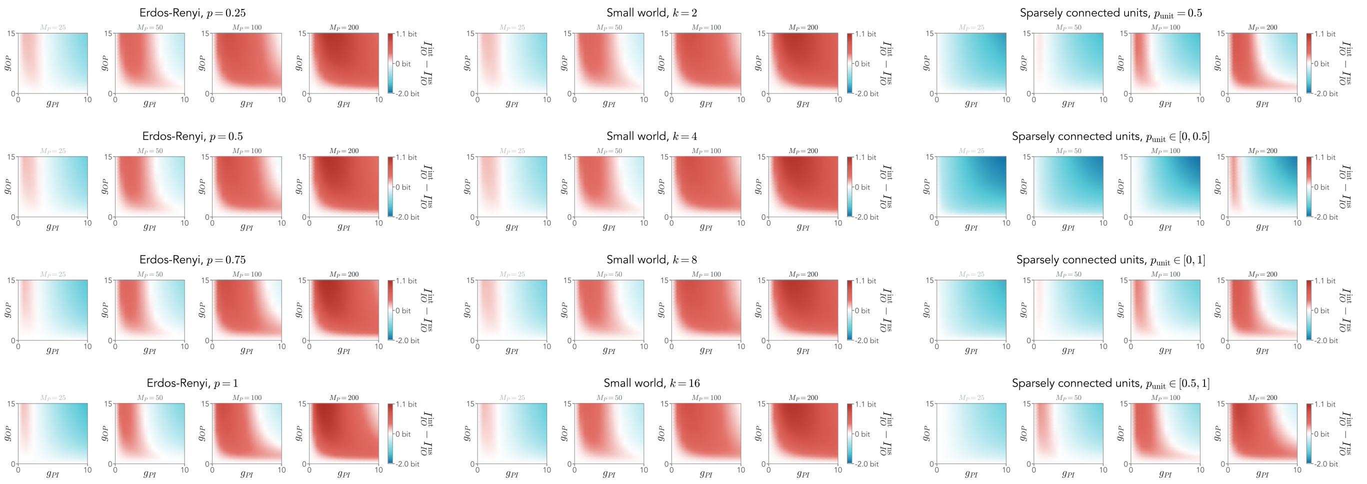


FIG. S6. Difference between the input-output mutual information of nonlinear integration and nonlinear summation, in the fast processing case. We compare different topologies of the processing unit and different sparsity of the connections between the units. For an Erdos-Renyi topology in the processing unit, a higher probability of connection $p$ tends to favor nonlinear summation at lower processing dimensions. A similar effect is present in a small world network when increasing the number of neighbors $k$. The case of a Barabasi-Albert network is reported in the main text. The opposite happens when connections between the units are sparse (right panels), both in the case where the probability of connection between the nodes of two units $p_{\text{unit}}$ is fixed, and when $p_{\text{unit}}$ is randomly extracted from a uniform distribution.

where $n_{\text{samples}}$ is the number of samples at hand, $q(x) = 3(n-1)^2/[(n-2)(n-3)]$, $s$ is the sample skewness,

$$s = \frac{1}{n_{\text{samples}}} \frac{\sum_i \left[x_O^{(i)} - \langle x_O \rangle\right]^3}{\left[\langle x_O^2 \rangle - \langle x_O \rangle^2\right]^{3/2}} \tag{S49}$$

and $\kappa$ is the excess kurtosis,

$$\kappa = \frac{1}{n_{\text{samples}}} \frac{\sum_i \left[x_O^{(i)} - \langle x_O \rangle\right]^4}{\left[\langle x_O^2 \rangle - \langle x_O \rangle^2\right]^{2}} - 3\,. \tag{S50}$$

We note that the bimodality coefficient can take values between 0 and 1, with $b = 1$ for a perfectly bimodal distribution such as the sum of two Dirac's delta functions centered at different points. It is also easy to check that, for a uniform distribution, we have $b = 5/9 \approx 0.55$. Hence, a value significantly higher than this may indicate a high degree of bistability. In Figure S7, we show how the bimodality coefficient of the output distribution behaves as a function of $M_I$ and $M_P$ in a system with a fast processing unit, as in the main text but for a broader parameter range. We find that both for nonlinear summation and integration smaller dimensions favor the bistability, as the small sizes of the random matrices at play favor the presence of more diverse elements. Crucially, nonlinear integration typically features a higher bimodality coefficient, suggesting that the sensitivity of the output distribution is enhanced by integration.

## A. Tuning the output bistability

So far, we have considered the nonlinearity implemented through a hyperbolic tangent centered in zero, i.e., with a functional form of the type $\tanh x$, in the two different settings of nonlinear summation and integration. To gain more insights into the origin of the bistable dynamics observed in the main text, here we add an internal parameter to the hyperbolic tangent shaping the interaction between the processing and the output unit. That is, we add a parameter $\theta$ tuning the saturation regimes by modulating its argument, i.e., $x \to x - \theta$. We detail how to incorporate this parameter into our calculations, leading to the fact that, as shown in the main text, the net effect of the presence of this additional parameter is to tune the height of the distribution peaks in the regimes in which the output presents an emergent bimodal distribution.

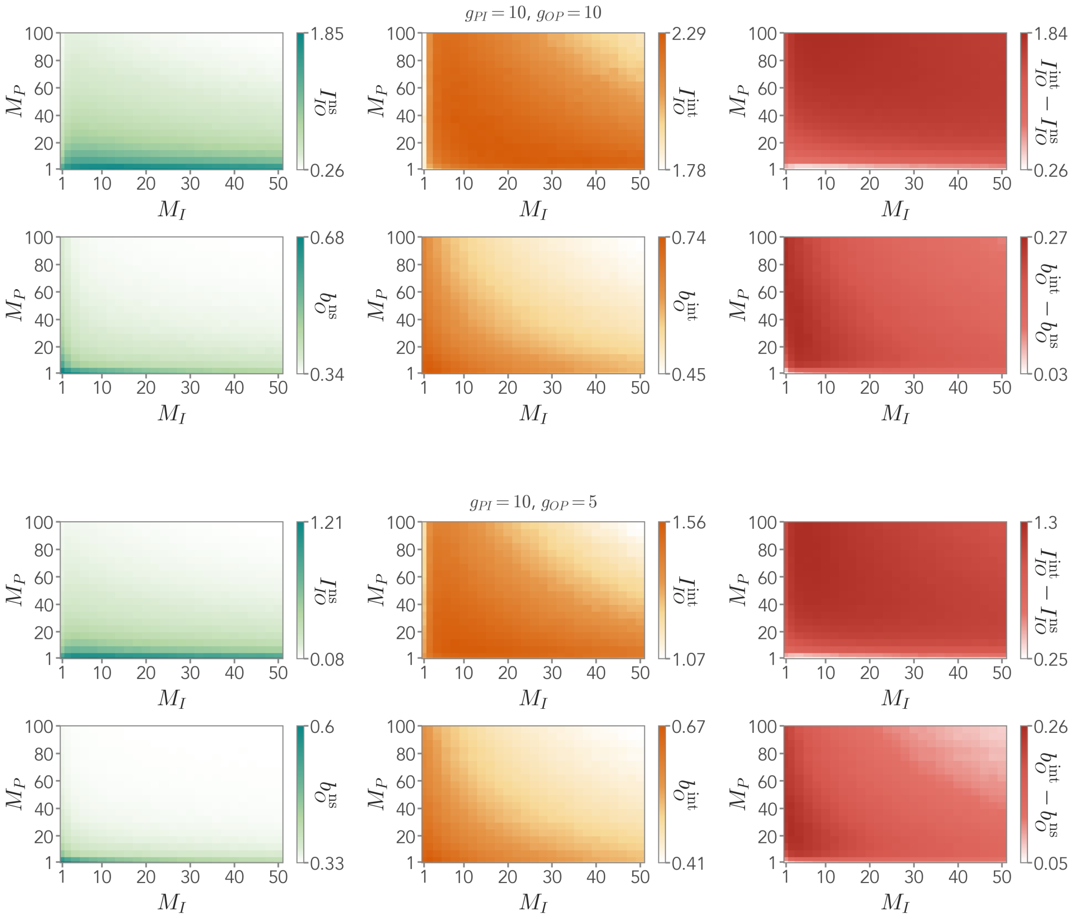


FIG. S7. Mutual information between the input and the output (first and third row) and bimodality coefficient of the output distribution (second and fourth row) for a system with a fast processing unit, with $\sigma_{OP} = \sigma_{PI} = 1$, $\sigma_I = \sigma_P = 0.9$, and $g_{PI} = g_{OP} = 10$ (top two rows) and $g_{PI} = 10$, $g_{OP} = 5$ (bottom two rows). For nonlinear integration (orange), information is higher at large $M_P$ for small input dimensions, and vice-versa. For nonlinear summation (teal), instead, information tends to decrease with the processing dimensionality. Both types of activation functions may display a bistable output distribution, particularly for smaller dimensions, although nonlinear integration enhances bimodality. All information is measured in bits. Results are averaged over $10^3$ realization of the random matrices. For each realization, $N_{\rm sam} = 2 \cdot 10^3$.

The case of slow processing does not require additional analysis, as the structure of the joint distribution is valid for any nonlinear mechanism (see Eq. (S42)). As such, the presence of additional parameters will not change this result. On the other hand, the solution in the case of fast processing is sensitive to the form of the nonlinearity under consideration due to its Gaussian average appearing in the effective distributions. Let us start with the nonlinear sum. By following the steps highlighted above, obtaining a solution for this scenario amounts to determining the effective Fokker-Planck operator, $\mathcal{L}_{\mathcal{O}|\mathcal{I}}^{\rm NS,eff}$, whose drift depends on

$$\begin{aligned}
\left\langle \phi_{OP}^i \right\rangle_P^{\rm ns}(\mathbf{x}_I) &\propto \int dx_P^1 \dots dx_P^j \dots dx_P^{M_P} \exp\left[-\frac{1}{2}(\mathbf{x}_P - \mathbf{m}_{P|I}(\mathbf{x}_I))^T \hat{\Sigma}_P^{-1} (\mathbf{x}_P - \mathbf{m}_{P|I}(\mathbf{x}_I))\right] \sum_{j=1}^{M_P} \frac{A_{OP}^{ij}}{M_P} \tanh\left(x_P^j - \theta_P^j\right) \\
&\propto \sum_{j=1}^{M_P} \frac{A_{OP}^{ij}}{M_P} \int dx_P^j \exp\left[-\frac{1}{2\Sigma_P^{jj}}\left(x_P^j - m_{P|I}^j(\mathbf{x}_I)\right)^2\right] \tanh\left(x_P^j - \theta_P^j\right)
\end{aligned}$$

where we neglected the normalization terms for brevity and, as before, exploited the fact that the hyperbolic tangent term depends on the components of $\mathbf{x}_P$ separately. Moreover, we allowed for the presence of as many internal

parameters $\theta_P^j$ as the number of processing states, for the sake of generality. For each $j$, this integral is of the form

$$\int_{-\infty}^{+\infty} dz \tanh(z-\theta) e^{-\frac{(z-m)^2}{2\sigma^2}} \xrightarrow{\zeta=z-\theta} \left[ \int_0^{+\infty} d\zeta \tanh(\zeta) e^{-\frac{(\zeta-(m-\theta))^2}{2\sigma^2}} - \int_0^{+\infty} d\zeta \tanh(\zeta) e^{-\frac{(\zeta+(m-\theta))^2}{2\sigma^2}} \right] .$$

It can be solved following the procedure outlined above and the result in Eq. (S24) will only present an average shifted by $\theta$. Putting all the elements together, we obtained the following drift of the effective operator:

$$m_{O|I}^{\mathrm{ns},i}(\mathbf{x}_I) = \frac{g_{OP}}{M_P} \sum_{j=1}^{M_I} \sum_{k=1}^{M_P} \left(A_O^{-1}\right)^{ij} A_{OP}^{jk} \Bigg[ \mathrm{erf}\left( \frac{m_{P|I}^k(\mathbf{x}_I) - \theta_P^k}{\sqrt{2\Sigma_P^{kk}}} \right) + \\ + \sum_{n=1}^{\infty} (-1)^n e^{2n^2 \Sigma_P^{kk}} \left[ V_n^+ \left( m_{P|I}^k(\mathbf{x}_I) - \theta_P^k, \Sigma_P^{kk} \right) - V_n^- (m_{P|I}^k(\mathbf{x}_I) - \theta_P^k, \Sigma_P^{kk}) \right] \Bigg] .$$

We now move to the case of nonlinear integration. In this scenario, the effective Fokker-Planck operator $\mathcal{L}_{\mathcal{O}|\mathcal{I}}^{\mathrm{INT,eff}}$ depends on the following integral:

$$\left\langle \phi_{OP}^i \right\rangle_P^{\mathrm{int}} (\mathbf{x}_I) \propto \int dx_P^1 \dots dx_P^{M_P} \exp\left[ -\frac{1}{2} (\mathbf{x}_P - \mathbf{m}_{P|I}(\mathbf{x}_I))^T \hat{\Sigma}_P^{-1} (\mathbf{x}_P - \mathbf{m}_{P|I}(\mathbf{x}_I)) \right] \tanh\left( \sum_{j=1}^{M_P} \frac{A_{OP}^{ij}}{M_P} (x_P^j - \theta_P^j) \right) .$$

By performing the same change of variable employed above and determined by the matrix in Eq. (S28), we obtain:

$$\left\langle \phi_{OP}^i \right\rangle_P^{\mathrm{int}} (\mathbf{x}_I) \propto \int dz^1, \dots, dz^{M_P - 1} dy_i \exp\left[ -\frac{1}{2} (\mathbf{z} - \hat{C}_i \mathbf{m}_{P|I}(\mathbf{x}_I))^T (\hat{C}_i \hat{\Sigma}_P \hat{C}_i^T)^{-1} (\mathbf{z} - \hat{C}_i \mathbf{m}_{P|I}(\mathbf{x}_I)) \right] \tanh(y_i - \Theta_i) \\ \propto \int_{-\infty}^{+\infty} dy_i \exp\left[ -\frac{1}{2 v_{\mathrm{int}}^i} \left( y_i - \frac{1}{M_P} \sum_{j=1}^{M_P} A_{OP}^{ij} m_{P|I}^j(\mathbf{x}_I) \right)^2 \right] \tanh(y_i - \Theta_i)$$

where $\Theta_i = \sum_{j=1}^{M_P} \theta_P^j (A_{OP}^{ij}/M_P)$ is a global tuning parameter that depends on all output-processing interactions. As above, this integral can be carried out by exploiting the expansion of the hyperbolic tangent leading to:

$$m_{O|I}^{\mathrm{int},i}(\mathbf{x}_I) = g_{OP} \sum_{j=1}^{M_I} \left(A_O^{-1}\right)^{ij} \Bigg[ \mathrm{erf}\left( \frac{m_{\mathrm{int}}^i(\mathbf{x}_I) - \Theta_i}{\sqrt{2 v_{\mathrm{int}}^i}} \right) + \\ + \sum_{n=1}^{\infty} (-1)^n e^{2n^2 v_{\mathrm{int}}^i} \left[ V_n^+ \left( m_{\mathrm{int}}^i(\mathbf{x}_I) - \Theta_i, v_{\mathrm{int}}^i \right) - V_n^- (m_{\mathrm{int}}^i(\mathbf{x}_I) - \Theta_i, v_{\mathrm{int}}^i) \right] \Bigg]$$

where $v_{\mathrm{int}}^i$ and $m_{\mathrm{int}}^i$ have been defined in Eqs. (S29) and (S30).

## SUPPLEMENTARY REFERENCES


[1] M. Lukoševičius and H. Jaeger, "Reservoir computing approaches to recurrent neural network training," *Computer Science Review*, vol. 3, no. 3, pp. 127–149, 2009.

[2] H. Sompolinsky, A. Crisanti, and H.-J. Sommers, "Chaos in random neural networks," *Physical review letters*, vol. 61, no. 3, p. 259, 1988.

[3] J. Kadmon and H. Sompolinsky, "Transition to chaos in random neuronal networks," *Physical Review X*, vol. 5, no. 4, p. 041030, 2015.

[4] R. Engelken, F. Wolf, and L. F. Abbott, "Lyapunov spectra of chaotic recurrent neural networks," *Physical Review Research*, vol. 5, no. 4, p. 043044, 2023.

[5] N. Maheswaranathan, A. Williams, M. Golub, S. Ganguli, and D. Sussillo, "Universality and individuality in neural dynamics across large populations of recurrent networks," *Advances in neural information processing systems*, vol. 32, 2019.

[6] L. N. Driscoll, K. Shenoy, and D. Sussillo, "Flexible multitask computation in recurrent networks utilizes shared dynamical motifs," *Nature Neuroscience*, vol. 27, no. 7, pp. 1349–1363, 2024.

[7] G. Nicoletti and D. M. Busiello, "Information propagation in multilayer systems with higher-order interactions across timescales," *Physical Review X*, vol. 14, no. 2, p. 021007, 2024.

[8] G. Nicoletti and D. M. Busiello, "Information propagation in gaussian processes on multilayer networks," *Journal of Physics: Complexity*, vol. 5, p. 045004, oct 2024.

[9] O. Vasicek, "A test for normality based on sample entropy," *Journal of the Royal Statistical Society Series B: Statistical Methodology*, vol. 38, no. 1, pp. 54–59, 1976.

[10] L. F. Kozachenko and N. N. Leonenko, "Sample estimate of the entropy of a random vector," *Problemy Peredachi Informatsii*, vol. 23, no. 2, pp. 9–16, 1987.

[11] C. Lu and J. Peltonen, "Enhancing nearest neighbor based entropy estimator for high dimensional distributions via bootstrapping local ellipsoid," in *Proceedings of the AAAI Conference on Artificial Intelligence*, vol. 34, pp. 5013–5020, 2020.

[12] R. M. May, "Will a large complex system be stable?," *Nature*, vol. 238, no. 5364, pp. 413–414, 1972.

[13] G. Barzon, D. M. Busiello, and G. Nicoletti, "Excitation-inhibition balance controls information encoding in neural populations," *Physical Review Letters*, vol. 134, no. 6, p. 068403, 2025.

[14] R. Pfister, K. A. Schwarz, M. Janczyk, R. Dale, and J. B. Freeman, "Good things peak in pairs: a note on the bimodality coefficient," *Frontiers in psychology*, vol. 4, p. 700, 2013.
